# Outcomes of primary graft failure in acute myeloid leukemia patients following unrelated transplantation with post-transplant cyclophosphamide: a study from the ALWP/EBMT

**DOI:** 10.1038/s41409-025-02726-8

**Published:** 2025-10-18

**Authors:** Arnon Nagler, Jacques-Emmanuel Galimard, Sarah Kayser, Alexander Kulagin, Didier Blaise, Elena Parovichnikova, Jurjen Versluis, Maija Itäla-Remes, Goda Choi, Rodrigo Martino Bufarull, Simona Sica, Mieke W. H. Roeven, Peter von dem Borne, Ali Bazarbachi, Jaime Sanz, Mohamad Mohty, Fabio Ciceri

**Affiliations:** 1 Division of Hematology, Sheba Medical Center, Tel Hashomer, Israel; 2 EBMT Paris Study Unit; Department of Haematology, Saint Antoine Hospital; INSERM UMR 938, Sorbonne University, Paris, France; 3 Department of Hematology, Oncology and Cancer Immunology, Charité - Universitätsmedizin Berlin, corporate member of Freie Universität Berlin and Humboldt-Universität zu Berlin, Berlin, Germany; 4 RM Gorbacheva Research Institute, Pavlov University, Petersburg, Russian Federation; 5 Programme de Transplantation &Thérapie Cellulaire, Marseille, France; 6 National Research Center for Hematology, Moscow, Russian Federation; 7 Erasmus MC Cancer Institute, Rotterdam, The Netherlands; 8 Turku University Hospital, Turku, Finland; 9 University Medical Center Groningen (UMCG), Groningen, The Netherlands; 10 Hospital Santa Creu i Sant Pau, Barcelona, Spain; 11 Universita Cattolica S. Cuore, Rome, Italy; 12 Radboud University Medical center, Nijmegen, The Netherlands; 13 Leiden University Medical Center, Leiden, The Netherlands; 14 Hematology-Oncology Division, Department of Internal Medicine, American University of Beirut Medical Center, Beirut, Lebanon; 15 Hematology Department, Hospital Universitari i Politècnic La Fe, Valencia Departament de Medicina Universitat de Valencia, CIBERONC, Instituto Carlos III, Madrid, Spain; 16 Department of Haematology, Sorbonne University, Saint Antoine Hospital, INSERM UMR 938, Paris, France; 17 Ospedale San Raffaele, Haematology and BMT, Milano, Italy

**Keywords:** Acute myeloid leukaemia, Translational research, Stem-cell therapies

## Abstract

We assessed pGF in 2497 AML patients undergoing HSCT from 8-10/10 HLA-matched UD with PTCy. pGF was defined as failure to achieve an ANC ≥ 0.5 × 10^9^/L by day +30 after HSCT. The day +30 cumulative incidence of ANC was 92.6% (95%CI: 91.5–93.6), and the incidence of death without ANC recovery was 1.8% (95% CI: 1.3%–2.3%), corresponding to 141 (5.6%) patients not achieving an ANC ≥ 0.5 × 10^9^/L by day +30. PB was the graft source in 89.4% of the patients, and 56% received reduced-intensity conditioning. 21 patients underwent a second HSCT (15 in the absence of ANC recovery and 6 after ANC recovery). 1-y NRM and RI post-pGF were 22.1% and 22.4%, respectively. 1-y LFS and OS post-pGF were 59% and 55.5%, respectively. ANC recovery evaluated as a time-dependent covariate, KPS ≥ 90, and being in CR at the time of HSCT were associated with improved OS. In conclusion, the incidence of pGF post-unrelated HSCT with PTCy was 5.6%. Of the patients who failed to engraft by day +30, 70.9% did so by day +60. A second transplant can save some of the patients with pGF.

## Introduction

Post-transplant cyclophosphamide (PTCy) based anti-graft-versus-host disease (GVHD) prophylaxis has proven to be highly effective in preventing GVHD and reducing rates of both GVHD and non-relapse mortality (NRM), leading to substantial improvement in GVHD-free, relapse-free survival, including in the unrelated setting [1–7]. Given these unprecedented results, PTCy is increasingly being used as GVHD prophylaxis post-unrelated donor allogeneic hematopoietic stem cell transplantation (UD-HSCT) for patients with acute myeloid leukemia (AML) [1–7]. PTCy is relatively safe, but it is not without cost; side effects and toxicities include a potentially increased risk of cardiac events, hemorrhagic cystitis, delayed immune reconstitution, and late infections [8–11]. As for engraftment, several studies indicate that this may be slower with PTCy compared to conventional anti-GVHD prophylaxis [1, 12–16].

Primary graft failure (pGF) is a rare but life-threatening complication of allogeneic HSCT [17]. It is usually categorized as lack of initial engraftment defined as absolute neutrophil count (ANC) <0.5 × 10^9^/L in the absence of relapse and combined with the lack of donor chimerism by day +28 after transplantation from either mobilized peripheral blood (PB) stem cells or bone marrow (BM) grafts and by day +42 in the case of cord blood transplantation [17]. The incidence of pGF ranges from 0.8% to 20%, depending on the various transplant-related variables, including donor type, cell source, cell dose, primary disease, and conditioning intensity [18–22]. Known risk factors for pGF are human leukocyte antigen (HLA)-mismatching, cord blood transplantation, recipient’s donor-specific (anti-HLA) antibodies, non-myeloablative conditioning (NMA), low dose of infused hematopoietic stem cells, T-cell depletion, and ABO-mismatched transplants, among others [18, 19, 22–27]. Historically, pGF has been a major concern in the haploidentical setting, particularly when BM and reduced intensity conditioning (RIC) is used [28]. As for PTCy, limited data exist on incidence and risk factors for pGF in HSCT with PTCy. From a theoretical point of view, PTCy upregulates T regulatory cells, leading to long-term immune tolerance [10, 29, 30], and thus the incidence of pGF should be low despite the broad HLA disparity. In contrast, some recent data indicate higher early mixed donor chimerism with PTCy [31] and as mentioned above, delayed neutrophil engraftment and even increased red blood cells and platelet transfusion requirements during the first 30 days after transplantation [32]. Regarding haploidentical HSCT with PTCy, the initial study from Luznik et al. reported a graft failure rate of 13% with PTCy following BM infusion and NMA [33]. The Acute Leukemia Working Party (ALWP) previously analyzed risk factors for pGF in AML patients undergoing haploidentical HSCT with PTCy. The incidence of pGF was 6%, which is lower than that reported in T-cell-depleted haploidentical HSCT [28, 34]. Factors independently associated with the risk of non-engraftment were: secondary AML, RIC, and BM grafts [34]. However, the incidence of pGF in the setting of unrelated HSCT with PTCy may differ and is largely unknown. We thus assessed the incidence of pGF post 8–10/10 HLA-matched UD-HSCT with PTCy in patients with AML using the registry data of the ALWP of the European Society for Blood and Marrow Transplantation (EBMT).

## Subjects and methods

### Study design and data collection

This was a retrospective, multicenter analysis. Data were provided by the registry of the ALWP of the EBMT. The EBMT is a non-profit, scientific society representing more than 600 transplant centers, mainly located in Europe, which are required to report all consecutive stem cell transplantations and follow-ups once a year. Data are entered, managed, and maintained in a central database. Since 1990, all patients have provided informed consent authorizing the use of their personal information for research purposes. The validation and quality control program includes verification of the computer print-out of the entered data, cross-checking with the national registries, and on-site visits to selected teams. The study was approved by the ALWP of the EBMT institutional review board and conducted according to the Declaration of Helsinki and Good Clinical Practice guidelines.

### Criteria for selection

To estimate the day 30 cumulative incidence of ANC recovery and associated impact factors, the eligibility criteria comprised adult patients ≥18 years of age with AML who underwent a first HSCT between 2010 and 2022 from an 8 to 10/10 HLA-matched UD with PTCy as GVHD prophylaxis. HSCTs from umbilical cord blood, siblings, and haploidentical donors were excluded. Pre-transplantation preparative regimens included both RIC and myeloablative conditioning (MAC). Patients had to have available information on neutrophil recovery. These inclusion criteria were met by 2497 patients.

For the main objective of this study, only patients with pGF (defined as ANC < 0.5 × 10^9^/L within 30 days after HSCT and only patients alive by day +30) were included, corresponding to 141 patients.

Data collected included recipient and donor characteristics, including age, gender, cytomegalovirus serostatus, Karnofsky performance status (KPS), clinical characteristics, including disease type, disease status, cytogenetic risk (European LeukemiaNet [ELN] 2022 cytogenetics classification), year of transplant, type of conditioning regimen, stem cell source, and GVHD prophylaxis regimen. The latter was defined as MAC or RIC, based on reports from individual transplant centers as per previously established criteria [35], and regimens for GVHD prophylaxis were per institutional protocols. Grading of acute (a) GVHD was performed using established criteria [36]. Chronic (c) GVHD was classified as limited or extensive according to published criteria [37]. For this study, all necessary data were collected according to the EBMT guidelines, using the EBMT minimum essential data forms. The list of institutions contributing data to this study is provided in the Supplementary Appendix.

### Statistical analysis

In a first step, the primary endpoint was neutrophil recovery, defined as achieving an ANC ≥ 0.5 × 10^9^/L for 3 consecutive days on the full cohort of 2497 patients from HSCT. Cumulative incidence of day 30 ANC recovery was calculated, as well as the day 30 incidence of death without ANC recovery. Both outcomes were calculated using the cumulative incidence function and were mutually competing events. In order to evaluate the impact factor of day 30 ANC recovery and day 30 death without ANC recovery, these two outcomes were censored at day 30. In the second step, including the 141 patients with pGF and from day +31, the primary endpoint was ANC recovery as previously defined, and the secondary endpoints included the incidence of aGVHD, cGVHD, overall survival (OS), leukemia-free survival (LFS), relapse incidence (RI), and NRM.

Median follow-up was calculated by the reverse Kaplan–Meier method. OS was defined as the time to death from any cause. LFS was defined as survival with no evidence of relapse or progression. NRM was defined as death from any cause without previous relapse or progression. The probabilities of OS and LFS were calculated using the Kaplan–Meier method [38]. Neutrophil recovery, aGVHD, cGVHD, RI, and NRM were estimated using cumulative incidence curves in a competing risk setting. Death and second HSCT were considered as competing risks for neutrophil recovery. To estimate the cumulative incidence of aGVHD and cGVHD, relapse and death were considered as competing events. Univariate analyses were performed using the log-rank test for OS and LFS, and Gray’s test for cumulative incidence. Multivariate analyses were conducted using the Cox proportional-hazards regression model [38]. The impact of ANC recovery post-pGF was evaluated as a time-dependent covariate. Results were expressed as hazard ratios (HRs) with 95% confidence intervals (CIs). All *p*-values were two-sided, with a type I error rate of 0.05. Statistical analyses were performed using R 4.0.2 (R Core Team, 2020). R: A language and environment for statistical computing. R Foundation for Statistical Computing, Vienna, Austria. URL https://www.R-project.org/ [39].

## Results

**Fig. 1:**
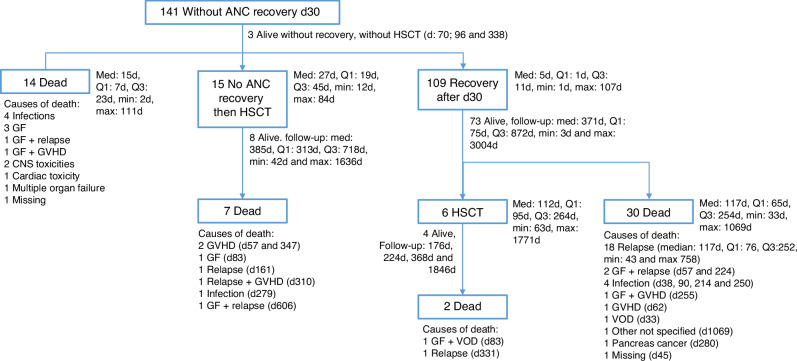
Hematopoietic stem cell transplantation (HSCT) outcomes of AML patients with primary graft failure following allogeneic stem cell transplantation from unrelated donors with post-transplant cyclophosphamide. The flow of the 141 patients that failed to achieve absolute neutrophil count (ANC) > 0.5 × 10^9^/L by day +30. ANC absolute neutrophil count, d day, HSCT hematopoietic stem cell transplantation, Med median, min minimum, max maximum, Q quarter, GF graft failure, CNS central nervous system, GVHD graft-versus-host disease, VOD Veno-occlusive disease.

In total, 2497 patients met the study inclusion criteria, of whom 141 (5.6%) patients failed to achieve ANC ≥ 0.5 × 10^9^/L by day +30 and were alive without second HSCT (Fig. 1). Table 1 shows the baseline demographic and clinical characteristics of these 141 patients. Median follow-up was 1.1 (95% CI 1–1.7) years post-pGF. The median year of the transplant was 2020 (IQR, 2017–2021). Median age was 56.4 (IQR, 46.2–64.3) years, and 61.7% were male. Disease status at HSCT was first or second complete remission in 65.7% and 8.6%, respectively, primary refractory or relapse in 21.4%, and “other” in 4.3% of the patients. Cytogenetic risk (ELN2022) was intermediate in 61.8%, adverse in 30.9%, and favorable in 7.3% of the patients, respectively, but data were missing for 31 (22%) of patients. Donors were 10/10 HLA-matched UD (50.1%) or 8-9/10 HLA-mismatched UD (36.8%), and 17 (12%) patients had missing data for this factor. KPS was ≥90 in 64.2% of the patients, and 80.4% were CMV seropositive. PB was the most frequently used stem cell source (89.4%). Female donor to male recipient was the combination used in 16.3% of patients. In vivo T cell depletion (TCD) with anti-thymocyte globulin was used in 14.9% of patients. Conditioning was RIC in 55.7% and MAC in 44.3% of patients (details of conditioning regimens are provided in Supplementary Table S1). The most frequent (29.8%) prophylaxis associated with PTCy was cyclosporine in combination with mycophenolate mofetil, followed by the latter in combination with tacrolimus (24.8%) of patients (Supplementary Table S2).

**Table 1:** Patient and disease characteristics of patients who failed to achieve ANC recovery by day +30.

Variables	Modalities	*N* = 141
Age at HSCT	Median (range)[IQR]	56.4 (18.2–74.1)
		[46.2–64.3]
Patient sex	Female	54 (38.3)
	Male	87 (61.7)
Year of HSCT	Median (range)[IQR]	2020 (2011–2022)
		[2017–2021]
Median FU (y)	−	1.1 [1–1.7]
ELN2022 cytogenetic risk	Favorable	8 (7.3)
	Adverse	34 (30.9)
	Intermediate	68 (61.8)
	Missing	31
Disease status at HSCT	CR1	92 (65.7)
	CR2	12 (8.6)
	CR ≥ 3	1 (0.7)
	PIF/Rel/Prog	30 (21.4)
	Other	5 (3.6)
	Missing	1
KPS	<90	49 (35.8)
	≥90	88 (64.2)
	Missing	4
Female to male	No	118 (83.7)
	Yes	23 (16.3)
Donor type	UD 10/10	72 (51)
	UD 9/10	48 (34)
	UD 8/10	4 (2.8)
	UD (missing HLA)	17 (12.1)
Patient CMV	Negative	27 (19.6)
	Positive	111 (80.4)
	Missing	3
Donor CMV	Negative	60 (42.9)
	Positive	80 (57.1)
	Missing	1
Source of cells	BM	15 (10.6)
	PB	126 (89.4)
Myeloablative conditioning	No	78 (55.7)
	Yes	62 (44.3)
	Missing	1
In vivo TCD	No	119 (84.4)
	ATG	21 (14.9)
	Campath	1 (0.7)

*HSCT* hematopoietic stem cell transplantation, *IQR* interquartile range, *FU* follow up, *y* years, *ELN* European leukemia net, *CR* complete remission, *CR1* first CR, *CR2* second CR, *CR≥3* third CR or CR more advanced, *KPS* Karnofsky performance status, *UD* unrelated donor, *HLA* human leukocyte antigen, *CMV* cytomegalovirus, *BM* bone marrow, *PB* peripheral blood, *TCD* T cell depletion; Unless otherwise stated, results are expressed as frequencies (%).

### Transplantation outcomes

**Fig. 2:**
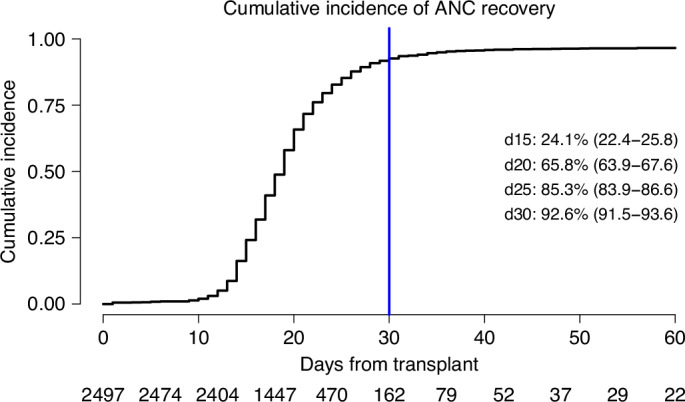
Hematopoietic stem cell transplantation (HSCT) outcomes of AML patients with primary graft failure following allogeneic stem cell transplantation from unrelated donors with post-transplant cyclophosphamide. Cumulative incidence of ANC recovery by day (d).

**Fig. 3:**
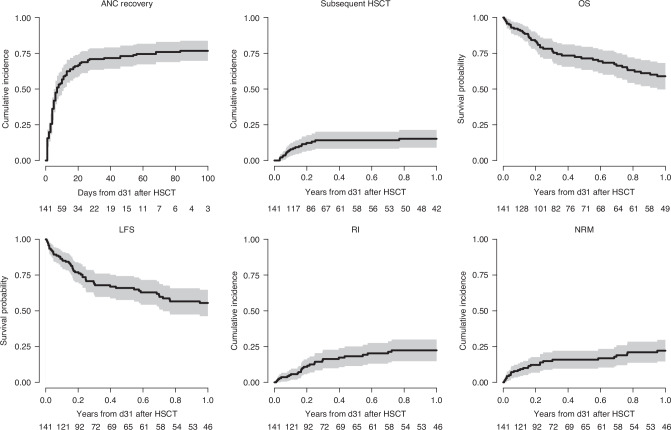
Hematopoietic stem cell transplantation (HSCT) outcomes of AML patients with primary graft failure following allogeneic stem cell transplantation from unrelated donors with post-transplant cyclophosphamide. Cumulative incidence of polymorphonuclear neutrophils (PMN) recovery, Cumulative incidence of second allogeneic transplantation (Subsequent HSCT), Overall survival (OS), leukemia-free survival (LFS); Relapse incidence (RI), Non-relapse mortality (NRM). PMN polymorphonuclear, HSCT hematopoietic stem cell transplantation, OS overall survival, LFS leukemia free survival, RI relapse incidence, NRM non relapse mortality.

Myeloid engraftment (ANC ≥ 0.5 × 10^9^/L) by day +30 post HSCT was 92.6% (95% CI, 62.6–77.7%), incidence of death within 30 days without ANC recovery was 1.8% (95%CI: 1.3–2.3%). In total, 141 patients (5.6%) did not achieve ANC recovery by day +30 and were alive and free of second HSCT. Of these, 109 (77.3%) recovered within the 30 consecutive days (Fig. 2). Twenty-one patients (15.2%) underwent a second HSCT as a rescue procedure (Figs. 1 and 3), 15 without ANC recovery and 6 with (Supplementary Table S3). Incidence of aGVHD grades II–IV and III-IV at day +100 post pGF were 15.5% (95% CI, 10–22%) and 3.6% (95% CI, 1.3–7.7%), respectively, while those of 1–year post pGF all grades and extensive cGVHD were 11.7% (95% CI, 6.1–19.1%) and 5.0% (95% CI, 1.8–10.6%), respectively (Table 2; Fig. 3). One-year NRM post pGF was 22.1% (95% CI, 15.1%–13.1%). A total of 53 patients died during the study period, including 14 in the absence of ANC recovery and subsequent HSCT (Table 3, Fig. 1). The original disease was the main cause of death in 41.2% of those who died, followed by GF in 21.5% (in whom about two-thirds had an accompanying contributing factor). Infection was the third most common cause of death (17.6%), and GVHD accounted for 5.9% of deaths. Other causes were infrequent (Table 3).

**Table 2:** Transplant outcomes.

Outcomes	Estimation (95%CI)
OS (1 y)	59 (49.1–67.5)
LFS (1 y)	55.5 (45.8–64.1)
RI (1 y)	22.4 (15.2–30.3)
NRM (1 y)	22.1 (15.1–30.1)
Second TX (1 y)	15.2 (9.6–22)
Poly recovery (30 d)	70.9 (62.6–77.7)
Poly recovery (60 d)	74.5 (66.4–80.9)
aGVHD-II/IV (100 d)	15.5 (10–22.2)
aGVHD-III/IV (100 d)	3.6 (1.3–7.7)
cGVHD (1 y)	11.7 (6.1–19.1)
cGVHD Ext (1 y)	5 (1.8–10.6)

*CI* confidence interval, *y* year, *d* day, *OS* overall survival, *LFS* leukemia-free survival, *RI* relapse incidence, *NRM* non-relapse mortality, *TX* transplantation, *aGVHD* acute graft-versus-host disease, *cGVHD* chronic graft-versus-host disease, *Ext* extensive, *Poly* polymorphonuclear neutrophils; Results expressed as percentages.

**Table 3:** Cause of death.

Causes of death	*N* = 53 (%)
Relapse	21 (41.2)
GF	11 (21.5)
Infection	9 (17.6)
GVHD	3 (5.9)
CNS toxicity	2 (3.9)
Other HSCT-related	2 (3.9)
VOD	1 (2)
Cardiac toxicity	1 (2)
Secondary malignancy	1 (2)
Missing	2

*GF* graft failure, *GVHD* graft-versus-host disease, *HSCT* hematopoietic stem cell transplantation, *VOD* veno-occlusive disease, *CNS* central nervous system; Results expressed as frequencies (%).

One-year RI post-pGF was 22.4% (95% CI, 15.2–30.3%). One-year LFS and OS post-pGF were 59% (95% CI, 45.8–64.1%) and 55.5% (95% CI, 49.1–67.5%), respectively (Table 2; Fig. 2). Table 4 summarizes the results of the univariate analysis. At 1 year, age ≥56 years (median age at HSCT), KPS < 90, female gender, and mismatched UD were significantly associated with an increased NRM. The only factor significantly associated with increased 1-year RI was not being in CR at the time of transplantation, which was also significantly associated with inferior 1-year LFS and OS. A KPS < 90 was also associated with inferior 1-year LFS, OS, and NRM, and RIC was significantly associated with inferior OS. PB grafts were associated with better day +30 ANC recovery post-pGF (Table 4).

**Table 4:** Univariate analysis of outcomes.

Variable	Modalities	d30 ANC recovery	1 y OS	1 y LFS	1 y RI	1 y NRM
Patient age group,	(18–56 years)	67.6 [55–77.5]	64.2 [49.8–75.4]	58.9 [44.8–70.6]	27.4 [16.4–39.6]	13.7 [6.2–24]
	(≥56–75 years)	74 [62.1–82.6]	54.3 [40.4–66.2]	52.4 [38.8–64.4]	17.7 [9.2–28.5]	29.8 [18.7–41.8]
	*P* value	0.17	0.17	0.38	0.21	0.02
Donor age group^*^	(17–28 years)	70.5 [57.1–80.4]	61.1 [45.4–73.6]	59.6 [44.2–72]	15.7 [7.5–26.5]	24.7 [13.2–38.1]
	(≥28–56 years)	72.4 [58.7–82.2]	55.2 [39.9–68]	49.9 [35–63.1]	28.7 [16.6–42]	21.4 [11.2–33.8]
	*P* value	0.9	0.9	0.761	0.383	0.636
KPS^^^	<90	57.1 [41.9–69.7]	39.4 [24.3–54.2]	35.7 [21.1–50.5]	30 [16.8–44.5]	34.3 [20.1–49]
	>= 90	78.4 [68.1–85.7]	69.8 [57.3–79.3]	66.1 [53.8–75.9]	17.5 [9.7–27.2]	16.4 [8.8–26]
	*P* value	0.06	<0.001	<0.001	0.142	0.003
Source of cells	BM	40 [15.6–63.7]	68.6 [35.9–87]	57.1 [24.3–80.2]	27.1 [5.1–56.4]	15.7 [2.2–40.9]
	PB	74.6 [66–81.3]	58.1 [47.6–67.1]	55.3 [45.1–64.4]	21.9 [14.5–30.3]	22.8 [15.2–31.3]
	*P* value	0.04	0.85	0.94	0.57	0.56
Patient sex	Male	74.7 [64–82.6]	63.1 [50.2–73.5]	60.5 [48.1–70.8]	24.8 [15.6–35.2]	14.7 [7.6–24]
	Female	64.8 [50.4–76]	52.8 [37.1–66.2]	48.5 [33.1–62.3]	17.9 [8.2–30.7]	33.5 [20.2–47.4]
	*P* value	0.19	0.14	0.22	0.47	0.02
Female to male recipient	No	71.2 [62.1–78.5]	62.1 [51.3–71.1]	58 [47.3–67.3]	20.4 [12.9–29]	21.6 [14–30.4]
	Yes	69.6 [45.3–84.7]	42.7 [20.1–63.7]	43 [21.2–63.2]	32.2 [13.5–52.7]	24.7 [8.2–45.8]
	*P* value	0.83	0.22	0.19	0.24	0.89
Disease status at HSCT^@^	CR	72.4 [62.7–80]	67.5 [56–76.7]	65.4 [54–74.6]	16.7 [9.5–25.7]	17.8 [10.6–26.6]
	Not CR	65.7 [47.1–79.1]	35.6 [18.9–52.8]	28.3 [13.4–45.1]	39.5 [22.5–56.1]	32.2 [16.1–49.6]
	*P* value	0.72	<0.001	<0.001	0.002	0.1
Patient CMV^#^	Negative	81.5 [58.8–92.4]	57.8 [34.9–75.1]	59.4 [36.7–76.3]	16.1 [4.7–33.6]	24.5 [9.2–43.6]
	Positive	68.5 [58.9–76.3]	59.1 [47.9–68.7]	54.3 [43.2–64]	24.6 [16.2–33.9]	21.1 [13.4–30.1]
	*P* value	0.35	0.83	0.74	0.34	0.62
Donor CMV^@^	Negative	68.3 [54.7–78.6]	49.1 [34.8–61.9]	45.2 [31.3–58]	29.3 [17.6–41.9]	25.6 [14.7–37.9]
	Positive	72.5 [61.1–81]	67.3 [53.7–77.7]	63.6 [50.1–74.3]	16.7 [8.7–27]	19.7 [10.8–30.5]
	*P* value	0.71	0.06	0.06	0.27	0.27
RIC	No	69.2 [57.6–78.3]	52.6 [38.6–64.9]	47.5 [33.6–60.1]	28.8 [17.4–41.3]	23.7 [13.8–35]
	Yes	74.2 [61.1–83.4]	65.7 [51.4–76.7]	63.6 [49.7–74.7]	16.9 [8.6–27.6]	19.5 [10.3–30.9]
	*P* value	0.45	0.04	0.07	0.29	0.28
Use of in vivo TCD	No	72.3 [63.2–79.4]	59.7 [48.8–69]	57.8 [47.2–67]	22.7 [14.9–31.4]	19.6 [12.3–28]
	Yes	63.6 [39.3–80.4]	55.2 [31–74]	44.6 [22.1–65]	20.5 [6–41]	34.9 [14.6–56.2]
	*P* value	0.17	0.5	0.29	0.9	0.18
Donor type^~^	MUD	77.8 [66.1–85.9]	66 [52.5–76.6]	62.8 [49.4–73.5]	21.6 [12.4–32.5]	15.6 [7.9–25.8]
	MMUD	61.5 [46.7–73.3]	48.9 [32.7–63.3]	43.6 [28.1–58.2]	25.5 [13.3–39.6]	30.9 [17.6–45.1]
	*P* value	0.07	0.06	0.1	0.77	0.03

^*^missing=22; ^**~**^missing=17; ^**^**^missing=4; ^**#**^missing=3, ^**@**^missing=1.

*d* day, *ANC* absolute neutrophil count, *y* year, *OS* overall survival, *LFS* leukemia free survival, *RI* relapse incidence, *NRM* non relapse mortality, *KPS* Karnofsky performance status, *BM* bone marrow, *PB* peripheral blood, *HSCT* hematopoietic stem cell transplantation, *CMV* cytomegalovirus, *RIC* reduced intensity conditioning, *CD* T cell depletion; Results expressed as percentage [95% CI].

Multivariate results are shown in Table 5. ANC recovery post pGF and KPS ≥ 90 was associated with improved 1-year OS (HR 0.46, 95% CI: 0.26–0.84, *p* = 0.01) and (HR 0.37, 95% CI: 0.21–0.65, *p* < 0.001), respectively, while not being in CR at the time of HSCT significantly decreased OS (HR 2.66, 95% CI: 1.50–4.71, *p* < 0.001).

**Table 5:** Multivariate analysis: impact of variables on overall survival.

Variable	Modalities	HR (95%CI)	*P* value
ANC recovery^a^	No	1	
	Yes	0.46 (0.26–0.84)	**0.01**
KPS	< 90	1	
	≥ 90	0.37 (0.21–0.65)	**< 0.001**
Age at HSCT		1.01 (0.92–1.12)	0.78
Disease status at HSCT	CR	1	
	Not in CR	2.66 (1.50–4.71)	**< 0.001**
Myeloablative conditioning	No	1	
	Yes	0.75 (0.42–1.33)	0.33

*HSCT* hematopoietic stem cell transplantation, *HR* hazard ratio, *CI* confidence interval, *ANC* absolute neutrophil count, *KPS* Karnofsky performance status, *CR* complete remission.

^a^ANC recovery evaluated as time-dependent covariate.

### Impact factors for day 30 absolute neutrophil count recovery

The impact factors for day 30 ANC recovery were evaluated (Supplementary Tables S4–6). Day 30 ANC recovery was positively affected by PB vs BM (1.60 (1.32–1.94), *p* < 0.001) and the use of in vivo TCD (HR 1.34,95% CI: 1.20–1.50, *p* < 0.001), while negatively affected by not being in CR at HSCT (HR 0.82,95% CI: 0.73–0.93, *p* = 0.002) and positive patient CMV serology (HR 0.91,95% CI: 0.83–0.99, *p* = 0.0047) (Supplementary Table S6). Death by day 30 in the absence of ANC recovery was negatively impacted by KPS < 90 (HR 2.02,95% CI: 1.08–3.79, *p* = 0.003) and not being in CR at HSCT (HR 3.35,95% CI: 1.79–6.27, *p* < 0.001) (Supplementary Table S6).

## Discussion

In this registry-based, real-life study assessing the incidence of pGF in a large cohort of AML patients who underwent UD transplantation with PTCy as GVHD prophylaxis, we demonstrated a rather low incidence (5.6%) of pGF; only 141 did not achieve ANC recovery by day +30. Of note, although 70.6% of the 141 patients engrafted by day +30, most of them recovered their myeloid counts by day +60. This observed incidence of pGF seems to be lower than the incidence previously reported for UD HSCT with calcineurin-based anti-GVHD prophylaxis [19–21, 26, 40–42]. The lower incidence may be due to improvement in HLA typing and donor selections, transplantation techniques and platforms, and pre-transplantation conditioning protocols, as well as due to novel anti-bacterial, viral, and fungal prophylaxis, GVHD prophylaxis, and better supportive care [43–45]. However, it is conceivable that the low incidence of pGF including in the mismatched UD HSCT cohort is due to the PTCy mechanism of action, impairing the proliferation of alloreactive T-cells, downregulating of pro-inflammatory cytokines, upregulating T regulatory cells, inducing T cell hypo-responsiveness and tolerance, combined with transcriptional exhaustion phenotype [10, 29, 30, 46–49].The low pGF we observed with PTCy is of paramount clinical importance as pGF is a lethal complication with a high mortality rate, higher than any other transplant-related complication [40, 50]. Of interest, the Center for International Blood and Marrow Transplant Research (CIBMTR) recently assessed the risk of late GF in adult patients with AML, acute lymphatic leukemia, or myelodysplastic syndrome (MDS) undergoing RIC haploidentical *versus* 8/8 UD-HSCT with PTCy, reporting similar 2-year incidences of 6.5% and 5.9%, respectively, indicating (as for the haploidentical HSCT) that the initial intense bidirectional alloreactivity between donor and recipient in haploidentical HSCT results in greater dysfunction and early engraftment failure but does not affect the graft long term [51]. However, the mortality rate was higher in the haploidentical HSCT (HR, 1.46; *p* = 0.007), with recurrent disease being the most common cause of death in both groups. Notably, none of the patients died of graft failure in the matched UD group, compared to 2% in the haploidentical group [51]. Recurrent disease was also the most common cause of death in our study, with GF being the second most frequent cause. GF is associated with a high mortality rate; we recently analyzed 243 patients with acute leukemia undergoing HSCT from various donors (~40% UD) who were complicated by pGF. We observed a 5-year NRM of 52% with infections being the main cause of mortality, in agreement with previous publications. pGF can lead to severe infections and hemorrhagic complications and is the most aggressive post-transplant problem, with extremely high overall mortality [27, 40, 41]. In the current study, infection was the third most frequent cause of death.

Our study is one of the first to focus on pGF in a homogeneous group of UD-HSCT recipients with PTCy in AML. One of the key findings is that about 77% of the patients who did not engraft by day +30 did so by day +60. This accords with the unique biology of PTCy-based anti-GVHD prophylaxis and the slower engraftment reported with PTCy, which is rather encouraging, and it differs from the kinetics seen with conventional anti-GVHD prophylaxis [12–16, 18–23]. This finding has major clinical implications for the timing of necessary salvage intervention and mainly salvage rescue transplantation, including from alternative donors [40, 52, 53]. Indeed, only 21 patients (15.2%) in our current study underwent a second HSCT.

Risk factors for pGF in HSCT with PTCy may differ from those previously reported with conventional-based GVHD prophylaxis. In our study, we observed better ANC recovery post-pGF with PB grafts, and an association of ANC recovery with OS in agreement with previous literature [18–21, 34, 40]. Other factors associated with improved OS were KPS and disease status at HSCT, as previously reported [40]. Recently, Mata et al. assessed pGF in 958 patients with various hematological malignancies receiving NMA HSCT with PTCy-based GVHD prophylaxis. Observed risk factors for pGF were age ≥65 years, an underlying diagnosis of MDS or myeloproliferative disorder (MPD), post-transplant viremia with human herpes virus-6, and low CD34+ and total nucleated cell dose (in those receiving BM grafts). Notably, pGF was not associated with HLA disparity [54]. The incidence of pGF was 3.8% and the 3-year NRM was 59% [54], both higher than we observed in a more homogeneous cohort of AML patients undergoing UD-HSCT, the majority (~90%) with PB grafts. The difference is most probably due to the many variances between the two studies, including factors known to affect the incidence of pGF, such as basic disease (higher incidence in MDS/MPD), type of donor (higher incidence with haploidentical donors), conditioning (higher incidence with NMA), and more. Concerning the risk of pGF in haploidentical-HSCT compared to UD-HSCT and the role of conditioning intensity, Gooptu et al. recently showed a significantly higher incidence of GF (11% vs. 3%, *p* < 0.001) after transplantation from haploidentical *versus* UDs in patients with acute leukemia or MDS when using PTCy and RIC in both groups, while no differences in GF were observed with MAC [55].

While not being the main objective of the study, the impact factors for day 30 ANC recovery were evaluated as well. Day 30 ANC recovery was positively affected by the graft source (PB vs BM) graft manipulation (the use of in vivo TCD), while negatively affected by not being in CR at the time of HSCT and positive patient CMV serology in agreement with previous publications [18, 19, 21, 26]. Moreover, as death in the absence of ANC recovery is the competing event of ANC recovery, it is important to mention that death by day 30 in the absence of ANC recovery was negatively impacted by KPS < 90 and not being in CR at the time of HSCT. As for additional risk factors for pGF, due to being a retrospective registry-based study, missing data such as cell dose, donor-specific antibody levels, early viral reactivation (e.g., HHV-6), and heterogeneity in some variables such as conditioning regimen, disease status, and HLA disparities, we could not define risk factors in our study. Other limitations of our study, being a retrospective registry design study, are the possibility that unavailable data, such as frontline therapies, molecular markers, measurable residual disease, or comorbidities, and center-level heterogeneity, might have influenced the outcomes.

However, in this real-life study, we observed a low incidence of pGF, which seems to be lower than that previously reported for UD HSCT with calcineurin-based anti-GVHD prophylaxis. Further studies aiming at improving engraftment, reducing the infection rate, and transplantation-related toxicity are warranted in an attempt to improve outcomes for leukemic patients with post-transplantation GF in the setting of PTCy.

## Supplementary information

Supplementary Appendix

## Data Availability

AN JEG, FC, and MM had full access to all study data (available upon data-specific request).
