# Supplementary material for: Outcomes of primary graft failure in acute myeloid leukemia patients following unrelated transplantation with post-transplant cyclophosphamide: a study from the ALWP/EBMT
Source: Bone Marrow Transplant. 2025 Oct 18;61(1):51–8. doi: 10.1038/s41409-025-02726-8 (PMC12819139; doi:10.1038/s41409-025-02726-8)
Supplement: Supplementary file 1 — Supplementary Appendix [file 41409_2025_2726_MOESM1_ESM.docx]

**Supplemental Table S1**. **Conditioning regimens**

| **Variable** | **Modalities** | **N=141 (%)** |
| --- | --- | --- |
| **Conditioning regimen** | BuCy | 2 (1.4) |
|  | BuFlu-based | 52 (36.9) |
|  | BuFluThio-based | 24 (17) |
|  | BuMel | 1 (0.7) |
|  | Cy+Flu+Thio | 1 (0.7) |
|  | FluMel-based | 5 (3.5) |
|  | TBI-based | 21 (14.9) |
|  | Treo+Flu | 17 (12.1) |
|  | Treo+Flu+Thio | 4 (2.8) |
|  | Treo+FluMel | 14 (9.9) |

**Abbreviations**: Bu-busulfan; Cy-cytoxan; Flu-fludarabine; Thio-thiotepa; Mel-melphalan; TBI-total body irradiation; Treo-treosulfan. Results expressed as frequencies (%)

**Supplemental Table S2**. **Graft-versus-host disease prophylaxis**

| **Variable** | **Modalities** | **N=141 (%)** |
| --- | --- | --- |
| **Additional GVHD prophylaxis** | CSA | 20 (14.2) |
|  | CSA+MMF | 42 (29.8) |
|  | MMF | 6 (4.3) |
|  | MMF+Siro | 15 (10.6) |
|  | MMF+Tacro | 35 (24.8) |
|  | MTX | 3 (2.1) |
|  | PTCy alone | 8 (5.7) |
|  | Tacro | 12 (8.5) |

**Abbreviations:** GVHD-graft-versus-host disease; CSA-cyclosporine; MMF-mycophenolate mofetil; Siro-sirolimus; Tacro-tacrolimus; MTX-methotrexate; PTCy-post-transplantation cyclophosphamide; Unless otherwise stated, results expressed as frequencies (%).

**Supplemental Table S3**: **Second Transplants- Patient and Transplant Characteristics**

*Same donor as previous HSCT

**Relapse but no ANC recovery

*** Patient reconstitution mentioned

**Abbreviations:** HSCT –hematopoietic stem cell transplantation; IQR- **interquartile** range; FU-follow up; y-years; ELN-EuropeanLeukemia net; CR- complete remission; CR1- first CR; CR2-second CR; CR>=3- third CR or CR more advanced; Rel-relapse; KPS – Karnofsky performance status; UD-unrelated donor; Haplo-Haploidentical; HLA-human leukocyte antigen; CMV-cytomegalovirus; BM-bone marrow; PB-peripheral blood; TCD-T cell depletion; Bu-busulfan; Cy-cytoxan; Flu-fludarabine; Thio-thiotepa; Mel-melphalan; TBI-total body irradiation; Treo-treosulfan; MAC-myeloablative; RIC-reduced intensity conditioning, GVHD-graft-versus-host disease, VOD – veno-occlusive disease, Unless otherwise stated, results are expressed as frequencies (%)

**Supplemental Table S4: Impact factors of d30 ANC recovery: Patient and Transplant Characteristics**

| **Variables** | **Modalities** | **All patients** | **ANC recovery within 30 days** | **No ANC recovery within 30 days and alive at d30** | **Dead within 30 days with ANC recovery** |
| --- | --- | --- | --- | --- | --- |
|  |  | **N=2497** | **N=2312** | **N=141** | **N=44** |
| Year of HCT | median [IQR] | 2019 [2017-2021] | 2019 [2017-2021] | 2020 [2017-2021] | 2019 [2016-2021] |
|  | (range) | (2010-2022) | (2010-2022) | (2011-2022) | (2010-2022) |
| Age at HCT | median [IQR] | 54.5 [42.3-63.7] | 54.2 [42-63.6] | 56.4 [46.2-64.3] | 60.8 [50.8-66.7] |
|  | (range) | (18-77.8) | (18-77.8) | (18.2-74.1) | (19.9-73.9) |
| Donor age | median [IQR] | 29.0 [23.9-36.5] | 29 [24-36.6] | 27.8 [23.4-36] | 31.9 [25.5-36.7] |
|  | (range) | (18-73.1) | (18-73.1) | (18.6-55.9) | (19.6-56.3) |
|  | missing | 234 | 213 | 16 | 5 |
| TNC | median [IQR] | 7.6 [5.6-9.8] | 7.6 [5.6-9.8] | 7.5 [5.8-9.4] | 8.5 [7.4-10.2] |
|  | (range) | (0-28) | (0-28) | (0.6-17.1) | (5.4-13.6) |
|  | missing | 1845 | 1711 | 98 | 36 |
| CD34 | median [IQR] | 6.0 [4.9-8] | 6 [4.9-8] | 5.7 [4.5-6.8] | 6.7 [5.6-7.7] |
|  | (range) | (0.1-60) | (0.1-41.5) | (0.6-60) | (4.9-12.2) |
|  | missing | 1700 | 1580 | 86 | 34 |
| KPS | < 90 | 646 (26.7) | 576 (25.7) | 49 (35.8) | 21 (48.8) |
|  | >= 90 | 1773 (73.3) | 1663 (74.3) | 88 (64.2) | 22 (51.2) |
|  | missing | 78 | 73 | 4 | 1 |
| Source of cells | BM | 138 (5.5) | 121 (5.2) | 15 (10.6) | 2 (4.5) |
|  | PB | 2359 (94.5) | 2191 (94.8) | 126 (89.4) | 42 (95.5) |
| Patient sex | Female | 1095 (43.9) | 1025 (44.4) | 54 (38.3) | 16 (36.4) |
|  | Male | 1399 (56.1) | 1284 (55.6) | 87 (61.7) | 28 (63.6) |
|  | missing | 3 | 3 | 0 | 0 |
| Donor sex | Female | 744 (30.2) | 689 (30.2) | 45 (31.9) | 10 (23.3) |
|  | Male | 1721 (69.8) | 1592 (69.8) | 96 (68.1) | 33 (76.7) |
|  | missing | 32 | 31 | 0 | 1 |
| Donor female to male recipient | No | 2130 (86.2) | 1975 (86.4) | 118 (83.7) | 37 (84.1) |
|  | Yes | 342 (13.8) | 312 (13.6) | 23 (16.3) | 7 (15.9) |
|  | missing | 25 | 25 | 0 | 0 |
| ELN2022 cyto | Favorable | 143 (7.1) | 134 (7.1) | 8 (7.3) | 1 (3.2) |
|  | Intermediate | 1395 (69.1) | 1311 (69.8) | 68 (61.8) | 16 (51.6) |
|  | Adverse | 482 (23.9) | 434 (23.1) | 34 (30.9) | 14 (45.2) |
|  | missing | 477 | 433 | 31 | 13 |
| Disease status | CR1 | 1685 (68) | 1576 (68.7) | 92 (65.7) | 17 (39.5) |
|  | CR2 | 352 (14.2) | 334 (14.6) | 12 (8.6) | 6 (14) |
|  | CR>=3 | 19 (0.8) | 18 (0.8) | 1 (0.7) | 0 (0) |
|  | CR (missing) | 26 (1) | 25 (1.1) | 0 (0) | 1 (2.3) |
|  | PIF/Rel/Prog | 345 (13.9) | 297 (12.9) | 30 (21.4) | 18 (41.9) |
|  | Other | 51 (2.1) | 45 (2) | 5 (3.6) | 1 (2.3) |
|  | missing | 19 | 17 | 1 | 1 |
| Disease status | CR | 2082 (84) | 1953 (85.1) | 105 (75) | 24 (55.8) |
|  | Non-CR | 396 (16) | 342 (14.9) | 35 (25) | 19 (44.2) |
|  | missing | 19 | 17 | 1 | 1 |
| Disease status with MRD | CR MRD neg | 577 (23.3) | 546 (23.8) | 25 (17.9) | 6 (14) |
|  | CR MRD pos | 384 (15.5) | 359 (15.6) | 23 (16.4) | 2 (4.7) |
|  | CR (missing MRD) | 1121 (45.2) | 1048 (45.7) | 57 (40.7) | 16 (37.2) |
|  | Non-CR | 396 (16) | 342 (14.9) | 35 (25) | 19 (44.2) |
|  | missing | 19 | 17 | 1 | 1 |
| Donor type | UD 10/10 | 1206 (48.3) | 1113 (48.1) | 69 (48.9) | 24 (54.5) |
|  | UD 10/10 (2 digits) | 3 (0.1) | 0 (0) | 3 (2.1) | 0 (0) |
|  | UD 9/10 | 690 (27.6) | 637 (27.6) | 43 (30.5) | 10 (22.7) |
|  | UD <=9/10 | 173 (6.9) | 162 (7) | 9 (6.4) | 2 (4.5) |
|  | UD (missing HLA) | 425 (17) | 400 (17.3) | 17 (12.1) | 8 (18.2) |
| Donor type | UD 10/10 | 1206 (48.3) | 1113 (48.1) | 69 (48.9) | 24 (54.5) |
|  | UD <=9/10 | 863 (34.6) | 799 (34.6) | 52 (36.9) | 12 (27.3) |
|  | UD (missing HLA) | 428 (17.1) | 400 (17.3) | 20 (14.2) | 8 (18.2) |
| Patient CMV | Negative | 690 (28.1) | 653 (28.7) | 27 (19.6) | 10 (23.3) |
|  | Positive | 1769 (71.9) | 1625 (71.3) | 111 (80.4) | 33 (76.7) |
|  | missing | 38 | 34 | 3 | 1 |
| Donor CMV | Negative | 1272 (51.7) | 1187 (52.1) | 60 (42.9) | 25 (56.8) |
|  | Positive | 1190 (48.3) | 1091 (47.9) | 80 (57.1) | 19 (43.2) |
|  | missing | 35 | 34 | 1 | 0 |
| Myeloablative  Conditioning | No | 1174 (47.3) | 1070 (46.6) | 77 (55) | 27 (62.8) |
|  | Yes | 1306 (52.7) | 1227 (53.4) | 63 (45) | 16 (37.2) |
|  | missing | 17 | 15 | 1 | 1 |
| Myeloablative conditioning and TBI | MAC Chemo | 1164 (46.9) | 1093 (47.6) | 55 (39.3) | 16 (37.2) |
|  | MAC TBI | 142 (5.7) | 134 (5.8) | 8 (5.7) | 0 (0) |
|  | RIC | 1174 (47.3) | 1070 (46.6) | 77 (55) | 27 (62.8) |
|  | missing | 17 | 15 | 1 | 1 |
| TBI | No | 2086 (83.5) | 1926 (83.3) | 120 (85.1) | 40 (90.9) |
|  | Yes | 411 (16.5) | 386 (16.7) | 21 (14.9) | 4 (9.1) |
|  | missing | 0 | 0 | 0 | 0 |
| Conditioning regimen | BuFlu based | 1010 (40.5) | 948 (41.1) | 52 (36.9) | 10 (22.7) |
|  | BuFluThio based | 442 (17.7) | 410 (17.8) | 24 (17) | 8 (18.2) |
|  | BuCy based | 100 (4) | 95 (4.1) | 2 (1.4) | 3 (6.8) |
|  | TBI based | 411 (16.5) | 386 (16.7) | 21 (14.9) | 4 (9.1) |
|  | Treo based | 318 (12.8) | 271 (11.7) | 35 (24.8) | 12 (27.3) |
|  | BuMel based | 4 (0.2) | 3 (0.1) | 1 (0.7) | 0 (0) |
|  | FluMel based | 165 (6.6) | 154 (6.7) | 5 (3.5) | 6 (13.6) |
|  | Other Bu based | 23 (0.9) | 22 (1) | 0 (0) | 1 (2.3) |
|  | Other | 20 (0.8) | 19 (0.8) | 1 (0.7) | 0 (0) |
|  | missing | 4 | 4 | 0 | 0 |
| In vivo TCD | No | 2016 (80.7) | 1863 (80.6) | 119 (84.4) | 34 (77.3) |
|  | Yes | 481 (19.3) | 449 (19.4) | 22 (15.6) | 10 (22.7) |
| GVHD prevention | CSA+MMF based | 789 (31.6) | 730 (31.6) | 42 (29.8) | 17 (38.6) |
|  | CSA based | 342 (13.7) | 318 (13.8) | 20 (14.2) | 4 (9.1) |
|  | CSA+MTX based | 68 (2.7) | 65 (2.8) | 0 (0) | 3 (6.8) |
|  | MMF+TACRO based | 634 (25.4) | 587 (25.4) | 35 (24.8) | 12 (27.3) |
|  | MMF | 90 (3.6) | 84 (3.6) | 6 (4.3) | 0 (0) |
|  | MMF+SIRO | 169 (6.8) | 148 (6.4) | 15 (10.6) | 6 (13.6) |
|  | MTX | 88 (3.5) | 85 (3.7) | 3 (2.1) | 0 (0) |
|  | TACRO/SIRO-based | 211 (8.5) | 198 (8.6) | 12 (8.5) | 1 (2.3) |
|  | Other combination | 7 (0.3) | 7 (0.3) | 0 (0) | 0 (0) |
|  | PTCY alone | 99 (4) | 90 (3.9) | 8 (5.7) | 1 (2.3) |
| Death within 30 days | Infection |  |  |  | 33 (75) |
|  | Other HSCT-related |  |  |  | 7 (15.9) |
|  | VOD |  |  |  | 3 (6.8) |
|  | GVHD |  |  |  | 1 (2.3) |

**Abbreviations:** HSCT –hematopoietic stem cell transplantation; IQR- **interquartile** range; FU-follow up; y-years; ELN-EuropeanLeukemia net; CR- complete remission; CR1- first CR; CR2-second CR; CR>=3- third CR or CR more advanced; Rel-relapse; PIF-primary induction failure, prog-progressive, KPS – Karnofsky performance status; UD-unrelated donor; Haplo-Haploidentical; HLA-human leukocyte antigen; CMV-cytomegalovirus; BM-bone marrow; PB-peripheral blood; TCD-T cell depletion; MRD-measurable residual disease, Bu-busulfan; Cy-cytoxan; Flu-fludarabine; Thio-thiotepa; Mel-melphalan; TBI-total body irradiation; Treo-treosulfan; MAC-myeloablative; RIC-reduced intensity conditioning, GVHD-graft-versus-host disease, VOD – veno-occlusive disease, CSA-cyclosporine; MMF-mycophenolate mofetil; Siro-sirolimus; Tacro-tacrolimus; MTX-methotrexate; PTCy-post-transplantation cyclophosphamide Unless otherwise stated, results are expressed as frequencies (%)

**Supplemental Table S5: Impact factors of d30 ANC recovery: Univariate analysis**

| **Variables** | **Modalities** | **d30 Death without ANC recovery** | **d30 ANC recovery** |
| --- | --- | --- | --- |
| Patient age (y) | (18,54] | 1 [0.5-1.7] | 93.6 [92.1-94.9] |
|  | (54,77] | 2.5 [1.8-3.5] | 91.6 [89.9-93] |
|  | P value | **0.004** | **0.005** |
| Donor age (235 missing) | (18,29] | 1.3 [0.8-2.1] | 92.7 [91-94] |
|  | (29,75] | 2.1 [1.4-3.1] | 92.8 [91.2-94.2] |
|  | P value | 0.13 | 0.88 |
| KPS (78 missing) | < 90 | 3.3 [2.1-4.8] | 89.2 [86.5-91.3] |
|  | >= 90 | 1.2 [0.8-1.8] | 93.8 [92.6-94.8] |
|  | P value | **0.001** | **0.01** |
| Source of cells | BM | 1.4 [0.3-4.7] | 87.7 [80.8-92.2] |
|  | PB | 1.8 [1.3-2.4] | 92.9 [91.8-93.8] |
|  | P value | 0.78 | **<0.001** |
| Donor type | UD 10/10 | 2 [1.3-2.9] | 92.3 [90.6-93.7] |
|  | UD <=9/10 | 1.4 [0.8-2.4] | 92.6 [90.6-94.1] |
|  | P value | 0.31 | 0.06 |
| Patient sex (3 missing) | Male | 2 [1.4-2.8] | 91.8 [90.2-93.1] |
|  | Female | 1.5 [0.9-2.3] | 93.6 [92-94.9] |
|  | P value | 0.307 | **0.045** |
| Donor sex (32 missing) | Male | 1.9 [1.3-2.7] | 92.5 [91.2-93.7] |
|  | Female | 1.3 [0.7-2.4] | 92.6 [90.5-94.3] |
|  | P value | 0.32 | 0.1 |
| Female to male (25 missing) | No | 1.7 [1.2-2.4] | 92.7 [91.5-93.7] |
|  | Yes | 2 [0.9-4] | 91.2 [87.7-93.8] |
|  | P value | 0.68 | 0.07 |
| Complete remission (19 missing) | CR | 1.2 [0.8-1.7] | 93.8 [92.7-94.8] |
|  | Non-CR | 4.8 [3-7.2] | 86.4 [82.6-89.4] |
|  | P value | **<0.001** | **<0.001** |
| CMV patient (38 missing) | Negative | 1.4 [0.7-2.6] | 94.6 [92.7-96.1] |
|  | Positive | 1.9 [1.3-2.6] | 91.9 [90.5-93] |
|  | P value | 0.48 | **0.03** |
| CMV donor (35 missing) | Negative | 2 [1.3-2.8] | 93.3 [91.8-94.6] |
|  | Positive | 1.6 [1-2.4] | 91.7 [90-93.1] |
|  | P value | 0.49 | 0.27 |
| Myeloablative conditioning (17 missing) | No | 2.3 [1.6-3.3] | 91.1 [89.4-92.6] |
|  | Yes | 1.2 [0.7-1.9] | 94 [92.5-95.1] |
|  | P value | **0.04** | **0.007** |

**Abbreviations:** d-day; ANC-absolute neutrophil count; y-year; KPS- Karnofsky performance status; CMV- cytomegalovirus; Results expressed as percentage [95% CI]

**Supplemental Table S6: Impact factors of d30 ANC recovery: Multivariate characteristics**

| **Variables** | **Modalities** | **d30 death without ANC recovery** | | **ANC recovery (censored as d30)** | |  |
| --- | --- | --- | --- | --- | --- | --- |
|  |  | **HR (95% CI)** | **p** | **HR (95% CI)** | **p** |  |
| Source of cells | BM | 1 |  | 1 |  |  |
|  | PB | 1.65 (0.39-6.95) | 0.5 | 1.60 (1.32-1.94) | **< 0.001** |  |
| KPS | >=90 | 1 |  | 1 |  |  |
|  | < 90 | 2.02 (1.08-3.79) | **0.03** | 0.94 (0.85-1.04) | 0.22 |  |
| Age (HR for 5y increment) | | 1.09 (0.96-1.24) | 0.18 | 0.99 (0.98-1.01) | 0.31 |  |
| Patient sex | Male | 1 |  | 1 |  |  |
|  | Female | 0.66 (0.34-1.26) | 0.21 | 1.05 (0.97-1.01) | 0.23 |  |
| Disease status | CR | 1 |  | 1 |  |  |
|  | Non-CR | 3.35 (1.79-6.27) | **< 0.001** | 0.82 (0.73-0.93) | **0.002** |  |
| CMV patient | Negative | 1 |  | 1 |  |  |
|  | Positive | 1.38 (0.65-2.90) | 0.4 | 0.91 (0.83-0.99) | **0.047** |  |
| In vivo TCD | No | 1 |  | 1 |  |  |
|  | Yes | 1.18 (0.54-2.58) | 0.68 | 1.34 (1.20-1.50) | **< 0.001** |  |
| Myeloablative regimen | No | 1 |  | 1 |  |  |
|  | Yes | 0.65 (0.33-1.28) | 0.21 | 1.09 (0.99-1.18) | 0.07 |  |

HR>1 means more ANC recovery

**Abbreviations:** HSCT; hematopoietic stem cell transplantation; HR-hazard ratio; CI - confidence interval**;** ANC-absolute neutrophil count; BM-bone marrow; PB-peripheral blood KPS- Karnofsky performance status; y-year, CMV- cytomegalovirus CR –complete remission; TCD-T cell depletion
